# Supplementary material for: Single cell proteomic analysis defines discrete neutrophil functional states in human glioblastoma
Source: Nat Commun. 2025 Dec 15;17:621. doi: 10.1038/s41467-025-67367-3 (PMC12816625; doi:10.1038/s41467-025-67367-3)
Supplement: Supplementary file 1 — Supplementary Information [file 41467_2025_67367_MOESM1_ESM.pdf]

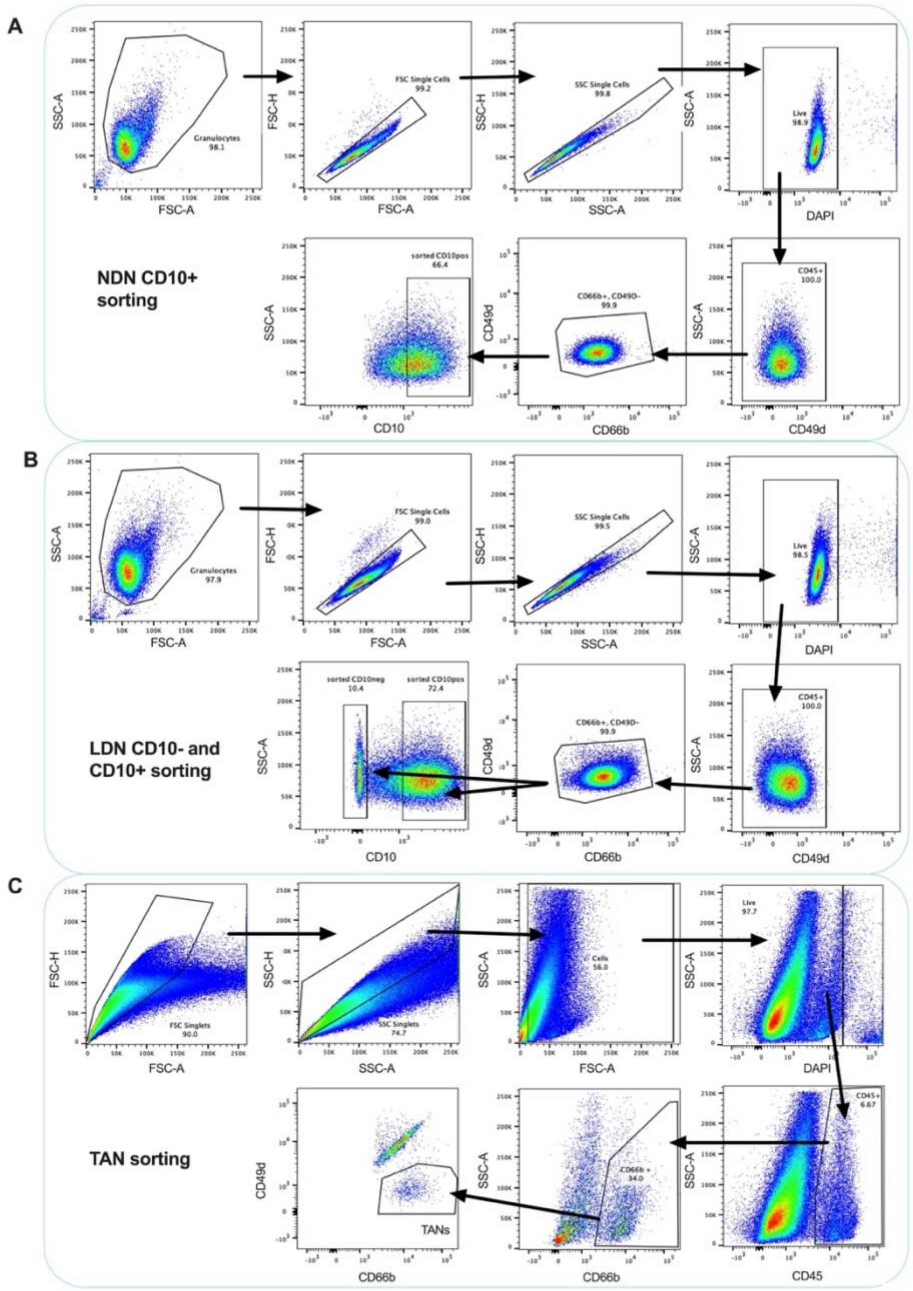

Supplementary Fig. 1 Gating strategy of neutrophils sorted from GBM blood and tumours

Representative dot plots illustrating the gating strategy of neutrophils sorted from circulating (**A**) glioblastoma (GBM) normal density neutrophils (NDN) (used for Fig. 1-3), (**B**) low density neutrophils (LDN) (used for Fig. 2-3) and (**C**) tumours associated neutrophils (TANs) post enzymatic digestion (used for Fig. 2-6). 500 cells GBM NDN CD10+, LDN CD10-, LDN CD10+ and TAN subpopulations in addition to healthy control NDN (n=6 for each subpopulation) were sorted into 384 well plates for the mini-bulk proteomics. For SCP analyses, single TANs were sorted into 384 well plates (n=330). NDN were gated on live CD45+, CD66b+, CD49-, CD10+ and LDN neutrophils were gated on live CD45+, CD66b+, CD49-,CD10- (immature) and live CD45+, CD66b+, CD49d-, CD10+ (mature) cells whereas TANs were gated on live CD45+, CD66b+, CD49d- for both mini-bulk and SCP analysis.

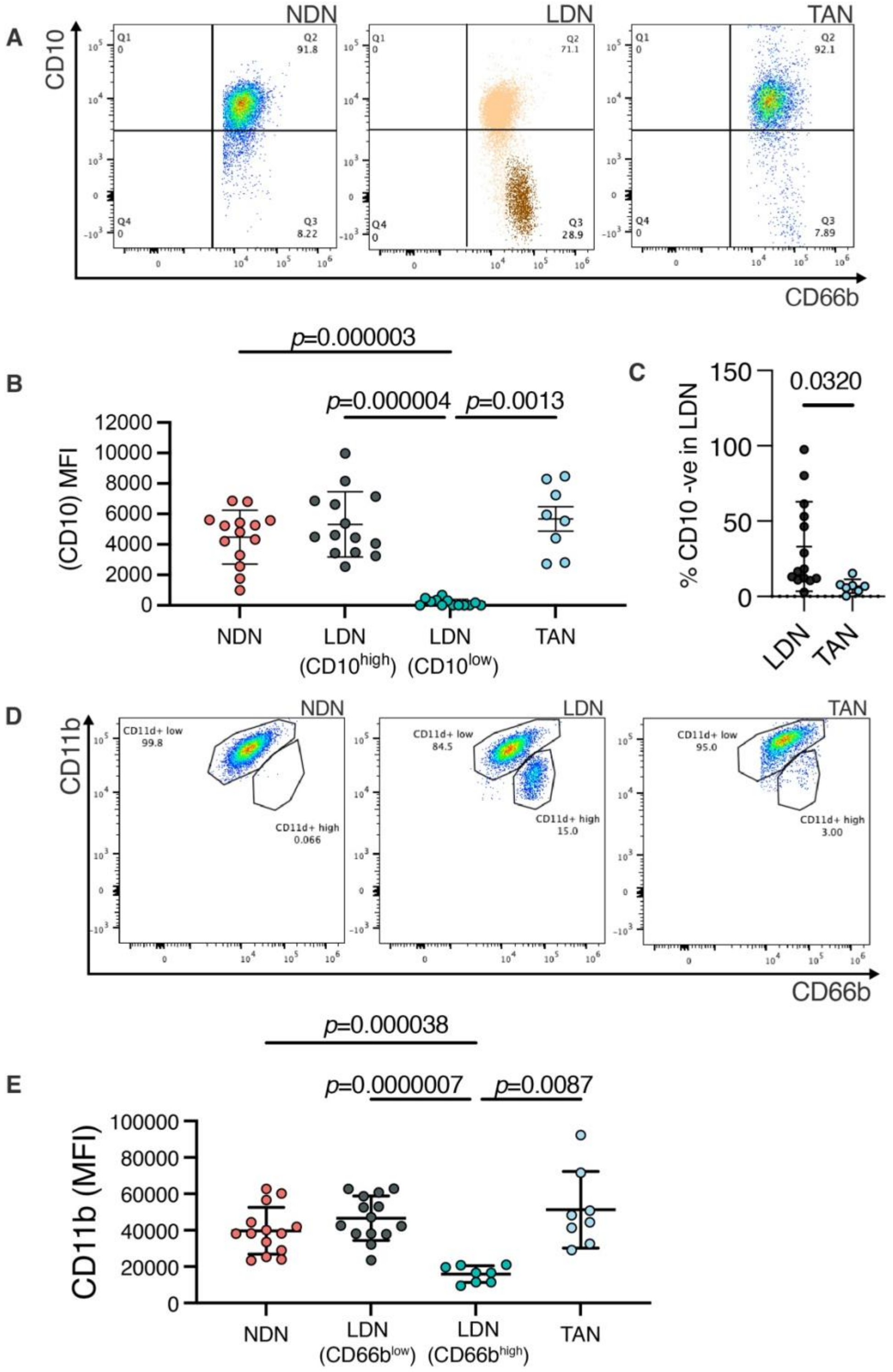

Supplementary Fig. 2 Neutrophil expansion and presence of an immature circulating neutrophil population

(A) Representative CD10 vs CD66b dot plots of circulating normal density neutrophils (NDN) (left), low density neutrophils (LDN) (middle) and tumour associated neutrophils (TAN) in glioblastoma (GBM, right). (**B**) Surface expression of CD10 expressed as median fluorescence intensity (MFI), NDN n=14, LDN CD10^{high} n=14, LDN CD10^{low} n=13, TAN n=8. (**C**) Percentage of cells with CD10- expression in LDN (n=15) and TAN (n=7). (**D**) Representative CD11b vs CD66b plots of circulating NDN, LDN and TAN in GBM gated on live, CD66b+, CD49d- expression. **(E)** Surface expression of CD11b expressed as MFI, NDN n=14, LDN CD10^{high} n=14, LDN CD10^{low} n=8, TAN n=8. Data represent mean ± SD. P-values were calculated with Welch's t-test (C), Brown-Forsythe and Welch ANOVA tests (B, E).

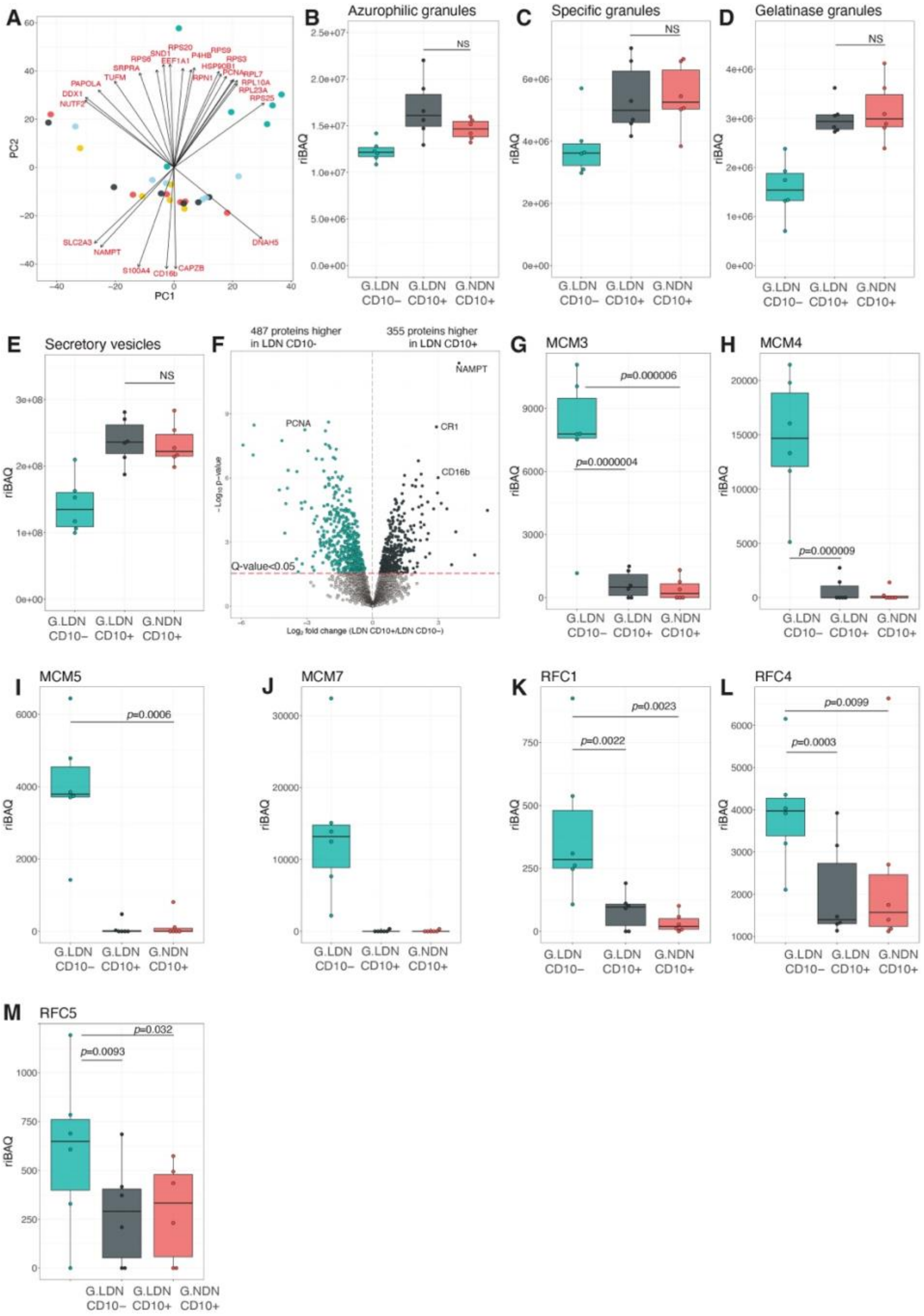

Supplementary Fig. 3 Granule and DNA replication proteins in mature (CD10+) and immature (CD10-) neutrophil proteomes (**A**) PCA plot showing the top 25 protein components for all mini-bulk samples. Boxplots (n=6 across all conditions) showing the riBAQ for **(B)** all azurophilic granules **(C)** all specific granules, **(D)** all gelatinase granules, **(E)** all secretory vesicles, **(F)** Volcano plot comparing CD10+ low density neutrophils (LDN) to CD10- low density neutrophils (LDN). Boxplots (n=6 across all conditions) showing the riBAQ for **(G)** Minichromosome Maintenance Complex Component 3 (MCM3), **(H)** Minichromosome Maintenance Complex Component 4 (MCM4), **(I)** Minichromosome Maintenance Complex Component 5 (MCM5), **(J)** Minichromosome Maintenance Complex Component 7 (MCM7), **(K)** Replication Factor C Subunit 1 (RFC1), **(L)** Replication Factor C Subunit 4 (RFC4) and **(M)** Replication Factor C Subunit 5 (RFC5) across LDN CD10-, LDN CD10+, normal density neutrophils (NDN) CD10+. For all boxplots the top and bottom hinges represent the 1st and 3rd quartiles. The top whisker extends from the hinge to the largest value no further than 1.5 × interquartile range (IQR) from the hinge; the bottom whisker extends from the hinge to the smallest value at most 1.5 × IQR of the hinge. All p-values calculated with limma using Empirical Bayes statistics for differential expression using a two-sided test. All volcano plots show the p-values and fold changes. All points above the red line have a q-value<0.05

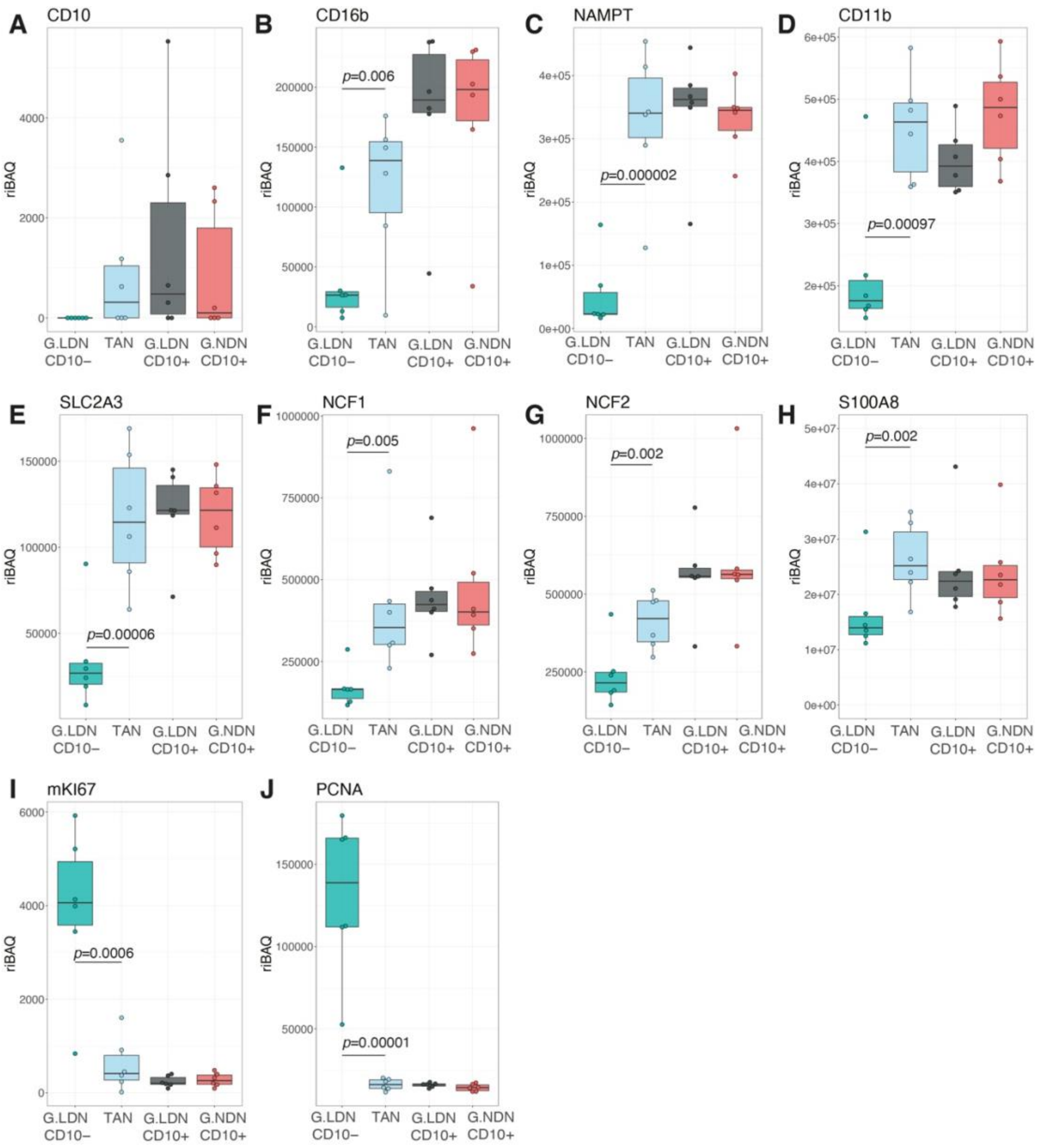

Supplementary Fig. 4 Maturity and proliferation markers across LDN CD10- ,TANs, LDN CD10+ and NDN CD10+

Boxplots (n=6 across all conditions) showing the riBAQ for **(A)** CD10 **(B)** CD16b, **(C)** Nicotinamide Phosphoribosyltransferase (NAMPT), **(D)** CD11b, **(E)** Solute Carrier Family 2 Member 3, GLUT 3 (SC2A3), **(F)** Neutrophil Cytosolic Factor 1 (NCF1), **(G)** Neutrophil Cytosolic Factor 2 (NCF2), **(H)** S100 Calcium Binding Protein A8 (S100A8), **(I)** Marker Of Proliferation Ki-67 (mKI67) and **(J)** Proliferating Cell Nuclear Antigen (PCNA) across low density neutrophils (LDN) CD10-, tumour associated neutrophils (TANs), LDN CD10+ and normal density neutrophils (NDN) CD10+. For all boxplots, the top and bottom hinges represent the 1st and 3rd quartiles. The top whisker extends from the hinge to the largest value no further than 1.5 × interquartile range (IQR) from the hinge; the bottom whisker extends from the hinge to the smallest value at most 1.5 × IQR of the hinge. All p-values were calculated with limma using Empirical Bayes statistics for differential expression using a two-sided test.

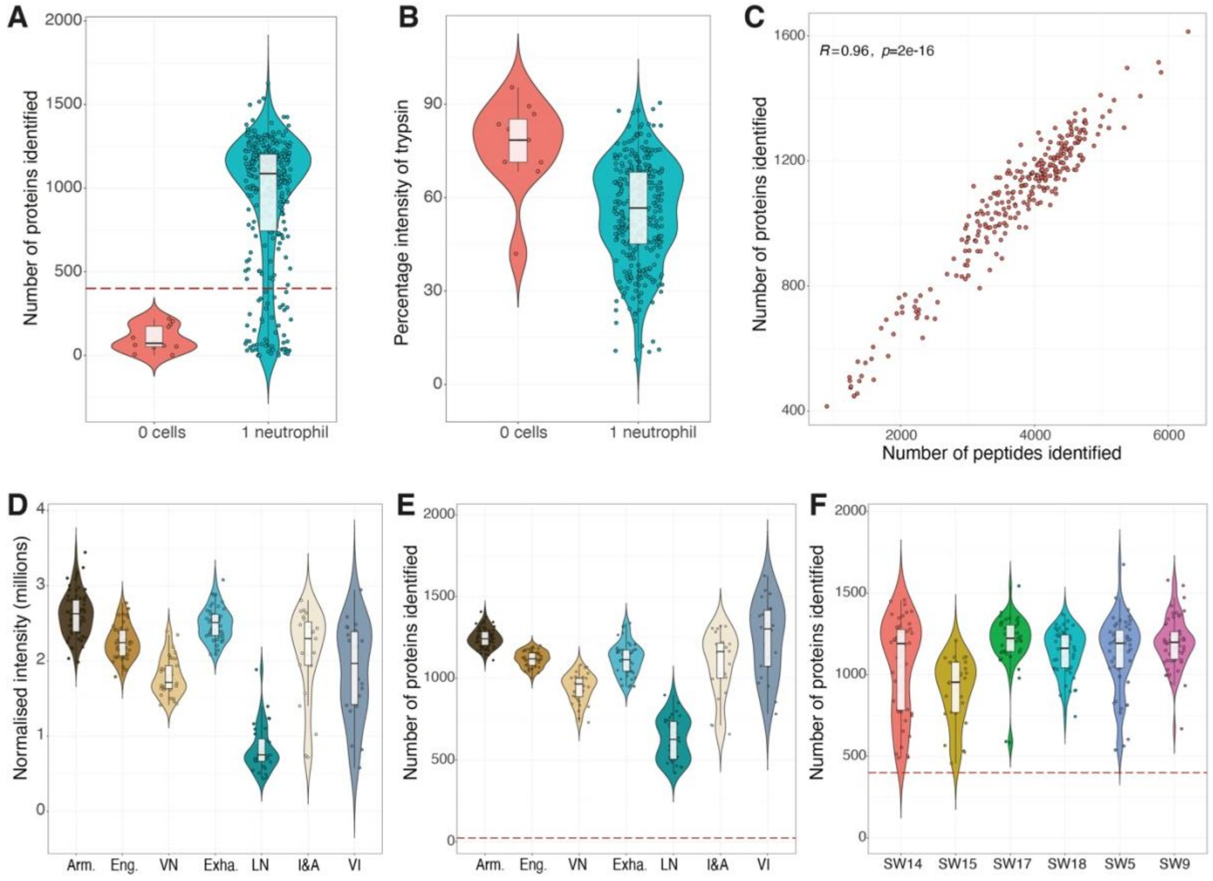

Supplementary Fig. 5 Single cell proteomics quality control

(A) Boxplots showing the number of proteins identified in the 0 cell (n=12) and the single neutrophil runs (n=330)**.** The red dotted line represents 400 proteins. **(B)** Boxplots showing the percentage of the total intensity represented by trypsin in the 0 cell (n=12) and the single neutrophil runs (n=330). **(C)** Scatter plot showing the number of proteins and peptides identified in each individual cell (n=277). Boxplots showing the **(D)** total normalized intensity and the **(E)** number of proteins identified across all 7 neutrophil clusters. Armed (Arm., n=76), Engaged (Eng., n=46), Vital NETs (VN, n=33), Exhausted (Exh., n=47), Lytic NETs (LN, n=32), Immunosuppressive and angiogenic (I&A, n=23) and Vascular Immature (VI, n=22). **(F)** Boxplot showing the number of proteins identified in each single neutrophil organized by patient. SW14(n=48), SW15(n=28), SW17(n=45), SW18(n=51), SW5(n=50), SW9(n=55). For all boxplots, the top and bottom hinges represent the 1st and 3rd quartiles. The top whisker extends from the hinge to the largest value no further than 1.5 × interquartile range (IQR) from the hinge; the bottom whisker extends from the hinge to the smallest value at most 1.5 × IQR of the hinge. P-values were calculated with a two-sided T test using the cor.test() function in the stats R package.

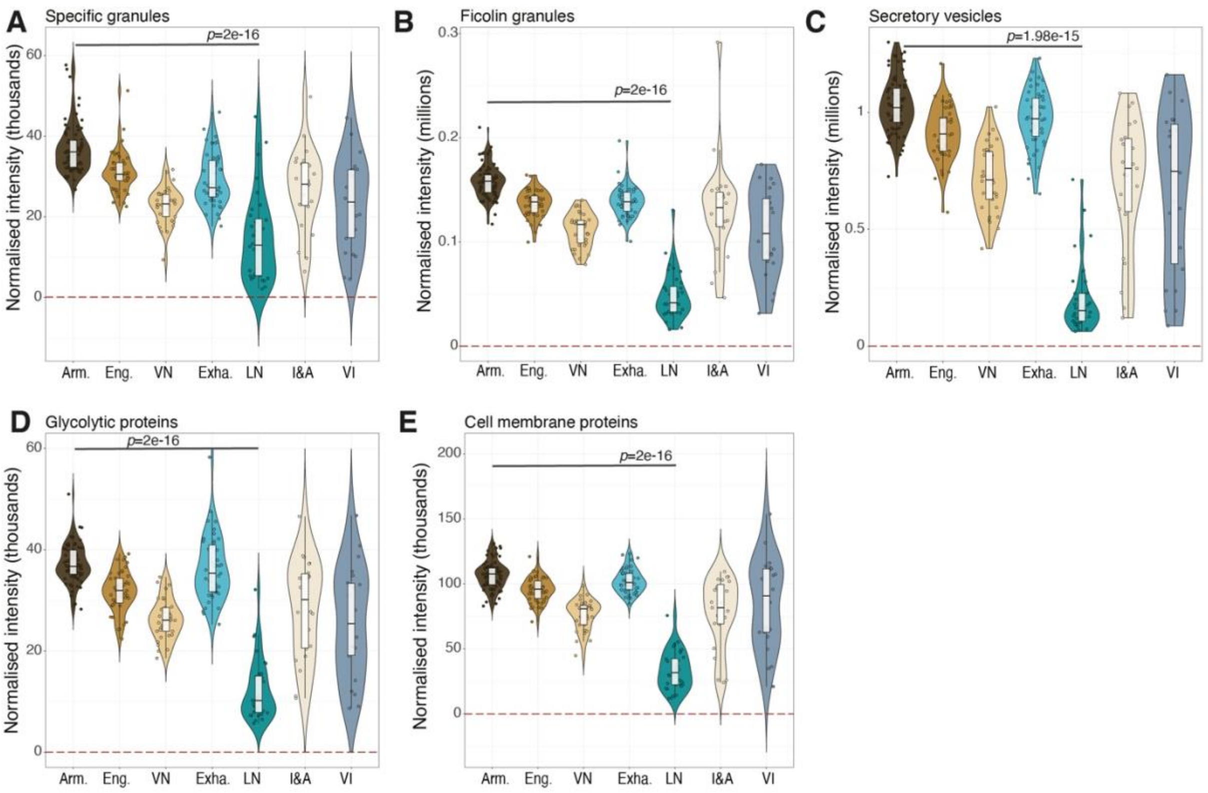

Supplementary Fig. 6 Lytic NETs reductions in granules, metabolism and cell surface proteins
Boxplots showing the sum of the normalized intensity for all proteins labelled as **(A)** specific granules, **(B)** ficolin granules, **(C)** secretory vesicles, **(D)** glycolytic proteins and **(E)** cell membrane proteins across all 7 neutrophil clusters. Across all boxplots Armed (Arm., n=76), Engaged (Eng., n=46), Vital NETs (VN, n=33), Exhausted (Exh., n=47), Lytic NETs (LN, n=32), Immunosuppressive and angiogenic (I&A, n=23) and Vascular Immature (VI, n=22). For all boxplots the top and bottom hinges represent the 1st and 3rd quartiles. The top whisker extends from the hinge to the largest value no further than 1.5 × interquartile range (IQR) from the hinge; the bottom whisker extends from the hinge to the smallest value at most 1.5 × IQR of the hinge. All p-values were calculated using a two-sided Welchs T test.
