# Supplementary material for: Single cell proteomic analysis defines discrete neutrophil functional states in human glioblastoma
Source: Nat Commun. 2025 Dec 15;17:621. doi: 10.1038/s41467-025-67367-3 (PMC12816625; doi:10.1038/s41467-025-67367-3)
Supplement: Supplementary file 2 — Description of Additional Supplementary Files [file 41467_2025_67367_MOESM2_ESM.pdf]

Description of Additional Supplementary Files

File Name: Supplementary Data 1.

Description: Bulk and mini-bulk proteomics processed data.

File Name: Supplementary Data 2.

Description: Single cell proteomics processed data.
