# Supplementary material for: Single cell proteomic analysis defines discrete neutrophil functional states in human glioblastoma
Source: Nat Commun. 2025 Dec 15;17:621. doi: 10.1038/s41467-025-67367-3 (PMC12816625; doi:10.1038/s41467-025-67367-3)
Supplement: Supplementary file 5 — Reporting Summary [file 41467_2025_67367_MOESM5_ESM.pdf]

nature portfolio

Corresponding author(s): Sarah Walmsley, Alejandro Brenes

Last updated by author(s): Nov 21, 2025

Reporting Summary

Nature Portfolio wishes to improve the reproducibility of the work that we publish. This form provides structure for consistency and transparency in reporting. For further information on Nature Portfolio policies, see our Editorial Policies and the Editorial Policy Checklist.

Statistics

For all statistical analyses, confirm that the following items are present in the figure legend, table legend, main text, or Methods section.

n/a	Confirmed	
☐	☒	The exact sample size (*n*) for each experimental group/condition, given as a discrete number and unit of measurement
☐	☒	A statement on whether measurements were taken from distinct samples or whether the same sample was measured repeatedly
☐	☒	The statistical test(s) used AND whether they are one- or two-sided<br>*Only common tests should be described solely by name; describe more complex techniques in the Methods section.*
☒	☐	A description of all covariates tested
☐	☒	A description of any assumptions or corrections, such as tests of normality and adjustment for multiple comparisons
☐	☒	A full description of the statistical parameters including central tendency (e.g. means) or other basic estimates (e.g. regression coefficient) AND variation (e.g. standard deviation) or associated estimates of uncertainty (e.g. confidence intervals)
☐	☒	For null hypothesis testing, the test statistic (e.g. F, t, r) with confidence intervals, effect sizes, degrees of freedom and P value noted<br>*Give P values as exact values whenever suitable.*
☒	☐	For Bayesian analysis, information on the choice of priors and Markov chain Monte Carlo settings
☒	☐	For hierarchical and complex designs, identification of the appropriate level for tests and full reporting of outcomes
☒	☐	Estimates of effect sizes (e.g. Cohen's *d*, Pearson's *r*), indicating how they were calculated

Our web collection on statistics for biologists contains articles on many of the points above.

Software and code

Policy information about availability of computer code

Data collection	Flow cytometry data was obtained using a BD LSRFortessa™ flow cytometer (Beckton Dickinson), with compensation performed using BD FACSDiva™ software version 8.0. The bulk proteomics data was acquired in DIA mode on an Orbitrap Exploris 480 (Thermo Scientific) coupled with an UltiMate 3000 RSLC nano (Thermo Scientific™). Mini-bulk and single cell proteomics data was obtained using a Vanquish Neo UHPLC (Thermo Fisher Scientific, #VN-S10-A-01) operated in trap-and-elute mode and coupled to an Orbitrap Astral mass spectrometer (Thermo Fisher Scientific, #BRE725600) equipped with a FAIMS Pro Duo interface (ThermoFisher Scientific, #OPTON-20068).
Data analysis	Flow cytometric data analysis was performed using FlowJo version 10.2. Bulk and mini-bulk proteomics data analysis was performed using Spectronaut 19.7 and the the single cell proteomic data analysis performed using Spectronaut 19.4. For all 3 searches the default parameters were altered to increase stringency; Precursor Qvalue Cutoff 0.01, Protein Qvalue Cutoff (experiment) 0.01, Protein Qvalue Cutoff (run) 0.01, Precursor PEP Cutoff 0.15, Protein PEP Cutoff 0.15, Protein LFQ Method Quant2.0, Quantity MS method (bulk) MS2, Quantity MS method (mini-bulk & single cell), MS1 Cross-Run Normalisation Off, Major (Protein) Grouping Protein Group ID, Major Group Quantity Peptide sum, Major Group Top N Off, Minor (Peptide) Grouping By Stripped Sequence, Minor Group Quantity Median precursor quantity, Minor Group Top N Off. All searches were performed using directDIA against a human SwissProt + isoforms database (November 2024) and an immune cell specific contaminant fasta file.The single cell proteomics data was analysed using Seurat v.5.2.1. For mini-bulk differential expression analysis was perform in R v4.5 using the Bioconductor package limma v3.64 Q values were calculated using the Bioconductor package qvalue v.4. CVs were calculated with the R package proteomicsCV v0.40. Statistics on the flowcytometry data were calculated using GraphPad Prism v10

For manuscripts utilizing custom algorithms or software that are central to the research but not yet described in published literature, software must be made available to editors and reviewers. We strongly encourage code deposition in a community repository (e.g. GitHub). See the Nature Portfolio guidelines for submitting code & software for further information.

Data

Policy information about availability of data

All manuscripts must include a data availability statement. This statement should provide the following information, where applicable:

- Accession codes, unique identifiers, or web links for publicly available datasets
- A description of any restrictions on data availability
- For clinical datasets or third party data, please ensure that the statement adheres to our policy

All proteomics data has been uploaded to PRIDE, part of the ProteomeXchange consortium. The bulk data is available under PXD061859, the mini-bulk data is available under PXD061052 and the single cell data under PXD061065. The processed proteomic data is available within the Immunological Proteome Resource.

Research involving human participants, their data, or biological material

Policy information about studies with human participants or human data. See also policy information about sex, gender (identity/presentation), and sexual orientation and race, ethnicity and racism.

Reporting on sex and gender	Male and female healthy control and Glioblastoma patients were enrolled in the study.
Reporting on race, ethnicity, or other socially relevant groupings	*Please specify the socially constructed or socially relevant categorization variable(s) used in your manuscript and explain why they were used. Please note that such variables should not be used as proxies for other socially constructed/relevant variables (for example, race or ethnicity should not be used as a proxy for socioeconomic status).*<br>*Provide clear definitions of the relevant terms used, how they were provided (by the participants/respondents, the researchers, or third parties), and the method(s) used to classify people into the different categories (e.g. self-report, census or administrative data, social media data, etc.)*<br>*Please provide details about how you controlled for confounding variables in your analyses.*
Population characteristics	*Describe the covariate-relevant population characteristics of the human research participants (e.g. age, genotypic information, past and current diagnosis and treatment categories). If you filled out the behavioural & social sciences study design questions and have nothing to add here, write "See above."*
Recruitment	Healthy control participants were recruited from the University of Edinburgh Centre for Inflammation Research Blood Resource. Exclusion criteria for healthy controls included the following: infection with any blood borne diseases, previous or current intravenous drug abuse, anaemia, blood clotting disorders, anticoagulant drug therapy, regular use of steroids and/or under the age of 16 years old. Patients with Glioblastoma were recruited and informed consent obtained by Clinical neurosciences, NHS Lothian staff.
Ethics oversight	These studies were approved by the Centre for Inflammation Research Blood Resource Management Committee (AMREC #15-HV-013) and Lothian NRS Bioresource, (East of Scotland Research Ethics Committee REC 1 #13/ES/0126; patients recruited between January 2015 to 2025 from NHS Lothian hospitals, Edinburgh, Scotland, UK).

Note that full information on the approval of the study protocol must also be provided in the manuscript.

Field-specific reporting

Please select the one below that is the best fit for your research. If you are not sure, read the appropriate sections before making your selection.

[x] Life sciences   [ ] Behavioural & social sciences   [ ] Ecological, evolutionary & environmental sciences

For a reference copy of the document with all sections, see nature.com/documents/nr-reporting-summary-flat.pdf

Life sciences study design

All studies must disclose on these points even when the disclosure is negative.

Sample size	No sample size calculations were performed to pre-determine samples size. Sample size was determined based on previous experience and pilot experiments in the Walmsley lab. The proteomics study used a total of 6 patients for mini-bulk and single cell analysis.
Data exclusions	One sample was excluded from the mini-bulk analysis as it had over 70% less proteins and peptides detected compared to all other 29 samples.
Replication	Experiments were replicated as detailed in the figure legends.
Randomization	All patients for single cell proteomics were GBM patients, hence no randomization was performed. However the samples when analysed on the mass spectrometer were randomly selected from the specific plates.
Blinding	All analysis was done on GBM cancer patients, however the researchers were blinded regarding the final classification of the different populations/clusters.

Reporting for specific materials, systems and methods

We require information from authors about some types of materials, experimental systems and methods used in many studies. Here, indicate whether each material, system or method listed is relevant to your study. If you are not sure if a list item applies to your research, read the appropriate section before selecting a response.

Materials & experimental systems

n/a	Involved in the study
☐	☒ Antibodies
☒	☐ Eukaryotic cell lines
☒	☐ Palaeontology and archaeology
☒	☐ Animals and other organisms
☒	☐ Clinical data
☒	☐ Dual use research of concern
☒	☐ Plants

Methods

n/a	Involved in the study
☒	☐ ChIP-seq
☐	☒ Flow cytometry
☒	☐ MRI-based neuroimaging

Antibodies

Antibodies used

Anti CD10 APC-Cy7 clone HI10a Biolegend (California, USA) Cat# 312212; RRID: AB_2146550; Lot# B275128
Anti CD11b PE-Cy7 clone ICRF44 Biolegend (California, USA) Cat# 301322; RRID: AB_830644; Lot# B247020
Anti CD45 BV421 clone HI30 Biolegend (California, USA) Cat# 304032; RRID: AB_2561357; Lot# B332800
Anti CD49d PerCP-Cy5.5 clone 9F10 Biolegend (California, USA) Cat# 304312; RRID: AB_10641699; Lot# B205823
Anti CD49d PE clone 9F10 Biolegend (California, USA) Cat# 304304; RRID: AB_314430; Lot# B250885
Anti CD66b APC clone G10F5 Invitrogen/ThermoFisher Scientific Cat# 17-0666-42; RRID: AB_2573152; Lot# 2252075
Anti CD66b FITC clone G10F5 Biolegend (California, USA) Cat# 305104; RRID: AB_314496; Lot# 2252075

Validation

Only commercially available antibodies were used in this study as per manufacturer's instructions with previous validation within Walmsley group in multiple other studies.

Plants

Seed stocks

Report on the source of all seed stocks or other plant material used. If applicable, state the seed stock centre and catalogue number. If plant specimens were collected from the field, describe the collection location, date and sampling procedures.

Novel plant genotypes

Describe the methods by which all novel plant genotypes were produced. This includes those generated by transgenic approaches, gene editing, chemical/radiation-based mutagenesis and hybridization. For transgenic lines, describe the transformation method, the number of independent lines analyzed and the generation upon which experiments were performed. For gene-edited lines, describe the editor used, the endogenous sequence targeted for editing, the targeting guide RNA sequence (if applicable) and how the editor was applied.

Authentication

Describe any authentication procedures for each seed stock used or novel genotype generated. Describe any experiments used to assess the effect of a mutation and, where applicable, how potential secondary effects (e.g. second site T-DNA insertions, mosiacism, off-target gene editing) were examined.

Flow Cytometry

Plots

Confirm that:

☒ The axis labels state the marker and fluorochrome used (e.g. CD4-FITC).

☒ The axis scales are clearly visible. Include numbers along axes only for bottom left plot of group (a 'group' is an analysis of identical markers).

☒ All plots are contour plots with outliers or pseudocolor plots.

☒ A numerical value for number of cells or percentage (with statistics) is provided.

Methodology

Sample preparation

Blood was drawn into S-Monovete® sodium citrate tubes (Sarstedt) or drawn into 50 mL polystyrene Falcon™ tubes containing 3.8% sodium citrate. Neutrophils were isolated from whole blood using the EasySep™ Direct Human Neutrophil Isolation Kit (StemCell Technologies). NDN and LDN cells were separated by Percoll gradient centrifugation. Following purification, cells were washed in FACS buffer (2% v/v HI-FBS in Dulbecco's phosphate buffered saline (DPBS) without Ca2+ or Mg2+) and stained with antibodies against CD66b, CD49d, CD10, CD11b. Immediately prior to analysis, DAPI was added to the cells. GBM tumour tissue was extracted and processed immediately by digestion to a single cell supsension in complete RPMI medium supplemented with 10% v/v HI-FBS (PAN-Biotech), 1% v/v penicillin-streptomycin (1000 U/L, Gibco), DNase

	(1000 U/L, Roche Diagnostics) and Collagenase Type IV (0.1% w/v, ThermoFisher Scientific) for 30 min at 37*C. Single cell suspension was then stained with antibodies against CD45, CD66B, CD49D, CD10. Immediately prior to analysis, DAPI was added to the sample.
Instrument	Cells were sorted on A FACS Aria Fusion sorter (BD) and data acquired using LSR Fortessa (BD).
Software	Flow cytometric data analysis was performed using FlowJo version 10.2.
Cell population abundance	Cell purity was determined using flow cytometry markers as detailed in the figure legends.
Gating strategy	Neutrophils were identified as:<br>Blood NDN: Mononuclear cells (FCS-A/SSC-A), Singlets (FSC-H/FSC-A, SSC-H/SSC-A), Live (DAPI negative),CD49d negative, CD66b positive, CD10positive.<br>Blood LDN: Mononuclear cells (FCS-A/SSC-A), Singlets (FSC-H/FSC-A, SSC-H/SSC-A), Live (DAPI negative),CD49d negative, CD66b positive, CD10 negative.<br>TAN:Singlets (FSC-H/FSC-A, SSC-H/SSC-A),Mononuclear cells (FCS-A/SSC-A), Live (DAPI negative), CD45 positive, CD66b positive, CD49d negative.

☒ Tick this box to confirm that a figure exemplifying the gating strategy is provided in the Supplementary Information.
